# CRF and urocortin peptides as modulators of energy balance and feeding behavior during stress

**DOI:** 10.3389/fnins.2014.00052

**Published:** 2014-03-18

**Authors:** Andreas Stengel, Yvette Taché

**Affiliations:** ^1^Division of General Internal and Psychosomatic Medicine, Charité Center for Internal Medicine and Dermatology, Charité-Universitätsmedizin BerlinBerlin, Germany; ^2^CURE: Digestive Diseases Research Center, Center for Neurobiology of Stress and Women's Health, Department of Medicine, Digestive Diseases Division at the University of California Los Angeles, and VA Greater Los Angeles Health Care SystemLos Angeles, CA, USA

**Keywords:** body weight, CRF, food intake, stress, urocortin

## Abstract

Early on, corticotropin-releasing factor (CRF), a hallmark brain peptide mediating many components of the stress response, was shown to affect food intake inducing a robust anorexigenic response when injected into the rodent brain. Subsequently, other members of the CRF signaling family have been identified, namely urocortin (Ucn) 1, Ucn 2, and Ucn 3 which were also shown to decrease food intake upon central or peripheral injection. However, the kinetics of feeding suppression was different with an early decrease following intracerebroventricular injection of CRF and a delayed action of Ucns contrasting with the early onset after systemic injection. CRF and Ucns bind to two distinct G-protein coupled membrane receptors, the CRF_1_ and CRF_2_. New pharmacological tools such as highly selective peptide CRF_1_ or CRF_2_ agonists or antagonists along with genetic knock-in or knock-out models have allowed delineating the primary role of CRF_2_ involved in the anorexic response to exogenous administration of CRF and Ucns. Several stressors trigger behavioral changes including suppression of feeding behavior which are mediated by brain CRF receptor activation. The present review will highlight the state-of-knowledge on the effects and mechanisms of action of CRF/Ucns-CRF_1/2_ signaling under basal conditions and the role in the alterations of food intake in response to stress.

## Introduction

Hans Selye was the first to introduce the biological concept and term of stress based on similar macroscopic changes namely the development of gastric erosions, involution of the lymphatic organs and hypertrophy of the adrenal glands in response to the exposure to a variety of noxious chemical agents or physical constraint in rats (Selye, 1936, 1976). Fourteen years later, Geoffrey Harris showed that various stressors induced the release of adrenocorticotropic hormone (ACTH) and provided the evidence for the release of a hypothalamic factor acting *via* hypophyseal portal vessels (De Groot and Harris, 1950; Harris, 1950). This concept was further supported by the purification of a hypothalamic factor stimulating ACTH release from the rat pituitary gland in 1955 (Guillemin and Rosenberg, 1955; Saffran et al., 1955). Therefore, this factor—which eluded its identification up to 1981—was named corticotropin-releasing factor (CRF) (Guillemin and Rosenberg, 1955; Saffran et al., 1955). From the beginning and with great foresight, Selye assumed that CRF would be “the first mediator that integrates the adaptive bodily response to stress” (Selye, 1976). After its identification and characterization by Vale's group as a 41 amino acid (aa) peptide (Vale et al., 1981), numerous studies unraveled pleiotropic stress-like actions induced by central injection of CRF beyond the mere activation of the hypothalamus-pituitary-adrenal gland axis. This includes the modulation of autonomic (sympathetic and sacral parasympathetic activation), visceral and immune functions but also behaviors (anxiogenic, reproductive and feeding) (Dunn and Berridge, 1990; De Souza, 1995; Heinrichs et al., 1997; Habib et al., 2000; Bale and Vale, 2004; Stengel and Taché, 2010).

It is well known that acute exposure to various stressors such as visceral (physical) (Stengel et al., 2011), immune (Basa et al., 2003; Wang et al., 2006; Stengel et al., 2010) and cognitive (psychological) (Rybkin et al., 1997; Kinzig et al., 2008; Calvez et al., 2011) stressors results in the suppression of food intake in rodents. This inhibitory effect is largely mediated by the recruitment of CRF signaling pathways in the brain. This is supported by the activation of CRF-containing neurons and pituitary ACTH release (Calvez et al., 2011; Wang et al., 2011a), the mimicry by brain injection of CRF or related peptides, urocortins, and its prevention by central injection of CRF receptor antagonists in rats (Koob and Heinrichs, 1999). Interestingly, models of chronic stress can have a dual effect on feeding and food preference in experimental animals and humans (Dallman, 2010). Social defeat stress led to an increase in daily food intake in mice giving rise to the recruitment of other/additional signaling systems under these conditions, while chronic exposure to a battery of physical/environmental stressors reduces food intake and body weight in rats (Lutter et al., 2008; Kumar et al., 2013).

The present review will describe first the state-of-knowledge on the distribution of the CRF signaling systems including CRF receptors, CRF, and urocortins in the brain and in the periphery and the effects and mechanisms of central and peripheral injection of CRF and Ucns on food intake. Lastly, the role of CRF receptor signaling pathways in the modulation of food intake under conditions of stress will be highlighted.

## The CRF signaling system

### Ligands: CRF and urocortins

CRF was identified in 1981 as a 41-aa hypothalamic peptide stimulating the release of ACTH and β-endorphin from the anterior part of the pituitary gland (Vale et al., 1981). Subsequently, other members of the CRF family were identified (Vaughan et al., 1995; Hsu and Hsueh, 2001; Lewis et al., 2001; Reyes et al., 2001), namely Ucn 1, a 40-aa peptide sharing 45% sequence identity with rat/human (r/h) CRF, Ucn 2, a 39-aa peptide sharing 34% homology with r/h CRF and 42% with r/h Ucn 1 (Reyes et al., 2001; Vaughan et al., 2013), and Ucn 3, a 38-aa peptide sharing only 26% homology with r/h CRF and 21% with r/h Ucn 1, respectively (Lewis et al., 2001). These four peptides are all derived from distinct genes highly conserved across mammalian, non-mammalian, and invertebrate species consistent with the physiological importance of this signaling system (Lovejoy and de Lannoy, 2013).

CRF is widely distributed in the rodent brain with robust expression at the mRNA and peptide level in the cerebral cortex, amygdala, hippocampus, paraventricular nucleus of the hypothalamus (PVN) and the Barrington's nucleus (Valentino et al., 1994; Wang et al., 2011a; Beckerman et al., 2013). Despite the fact that urocortins show an extensive brain distribution, little neuroanatomical overlap exists between CRF and urocortins. Ucn 1 has limited brain distribution with one major expression site which is the Edinger-Westphal nucleus (EWN) (Bittencourt et al., 1999; Morin et al., 1999; Shah et al., 2013), and to a lesser extent, the olfactory bulb, supraoptic nucleus (SON), ventromedial hypothalamus (VMH), lateral hypothalamic area, lateral superior olive, ambiguus nucleus, dorsolateral tegmental nucleus, linear and dorsal raphe nuclei, substantia nigra and cranial nerve motor nuclei, namely facial and hypoglossal (Kozicz et al., 1998; Bittencourt et al., 1999). Ucn 2 mRNA is found in the parvo- and magnocellular part of the PVN, the arcuate nucleus of the hypothalamus, SON, locus coeruleus, in cranial nerve motor nuclei (trigeminal, facial and hypoglossal nuclei) and also in the ventral horn of the spinal cord (Reyes et al., 2001; Mano-Otagiri and Shibasaki, 2004). It is important to note that the knowledge on Ucn 2's distribution at the peptide level has been limited by the lack of a specific Ucn 2 antibody. Lastly, Ucn 3 mRNA and peptide expression was detected in the amygdala, lateral septum, PVN, VMH, basomedial nucleus of the stria terminalis, dorsal raphe nucleus and in the area postrema (Lewis et al., 2001; Li et al., 2002; Mano-Otagiri and Shibasaki, 2004; Venihaki et al., 2004).

Besides the widespread expression of CRF and related peptides initially found in the central nervous system and thought to be restricted to this site, as commonly observed for other peptides, CRF and urocortins were also detected in visceral organs, namely the lung, heart, spleen, adipose tissue, gonads (Boorse and Denver, 2006; Wypior et al., 2011), pancreas (Li et al., 2003; van der Meulen et al., 2012) and the gastrointestinal tract (Kozicz and Arimura, 2002; Taché and Perdue, 2004; Taché and Bonaz, 2007; Yuan et al., 2012b) including the enteric nervous system (Liu et al., 2006; Kimura et al., 2007). The past decade witnessed an increasing knowledge on the peripheral expression and regulation of CRF and urocortin signaling systems and recognition of their implication in health and disease (Yuan et al., 2010; Buckinx et al., 2011; Overman et al., 2012; Diaz and Smani, 2013; Onorati et al., 2013).

### CRF receptors

CRF ligands bind to CRF_1_ and/or CRF_2_ receptors consisting of 415 aa and 411 aa, respectively, that are encoded from two distinct genes belonging to the B1 subfamily of seven-transmembrane G-protein coupled receptors encompassing seven membrane-spanning α-helices and an extracellular domain (Perrin and Vale, 1999). Both receptors share 70% identity within their species homologue. The most variable component is the binding domain that encompasses the N-terminal and the three extracellular coils that share only 40% homology between the two receptor subtypes. In their N-terminal extracellular region, CRF receptors contain several potential points of N-glycosylation along with several Cys residues that form disulfide bonds closely associated with their functionality (Zmijewski and Slominski, 2010; Liapakis et al., 2011). Various partial or total exon deletions or insertions in the CRF gene - in some cases associated with a shift in the open reading frame - generate multiple isoforms of CRF_1_ and CRF_2_ receptors (Pisarchik and Slominski, 2001; Wu et al., 2007, 2011; Zmijewski and Slominski, 2010). Among the nine human CRF_1a−i_ variants, CRF_1_, also named CRF_1a_, is the main functional receptor. The CRF_1b_ isoform is also called pro-CRF_1_, and this is the only variant coded by all 14 exons, leading to a 29-aa insertion into the first intracellular loop which impairs agonist binding and signaling activity compared to CRF_1a_ (Markovic and Grammatopoulos, 2010). The other isoforms CRF_1c−i_ have the exon 6 spliced out and in addition there are either cryptic exons (1h), or the skipping of single (1c, 1d, and 1f) or multiple exons (1e and 1g) (Pisarchik and Slominski, 2001, 2004; Wu et al., 2011). The latest splice variant identified, CRF_1i_ has a deletion of exon 4 and is functional. This was shown by the increased phosphorylation of the extracellular signal regulated kinase ½ (ERK1/2) in response to Ucn 1 assessed in CRF_1i_ transfected human embryonic kidney (HEK) cells (Wu et al., 2011). Other soluble isoforms such as CRF_1e_ and CRF_1h_ may play a modulatory role as their expression in transfected COS cells either decreased or amplified the CRF_1α_-coupled cAMP production induced by Ucn 1 (Pisarchik and Slominski, 2004).

With regards to the CRF_2_, three functional splice variants 2a, 2b, and 2c (also originally named 2α, 2β, and 2γ) derived from alternative splicing exist in humans, while in other mammals only two isoforms, CRF_2a_ and CRF_2b_ are expressed (Hauger et al., 2003; Hillhouse and Grammatopoulos, 2006). These isoforms differ structurally in their N-terminal extracellular domains (Hauger et al., 2003). The 34-aa N-terminal extracellular region of the CRF_2a_ is replaced by 61 aa in the CRF_2b_ and 20 aa in the CRF_2c_, while the C-terminus is common to all CRF_2_ receptor splice variants (Miyata et al., 2001; Dautzenberg et al., 2004; Wu et al., 2007). Sequence comparison indicated high homology between rat and mouse CRF_2a_ (94%) and mouse and human (92%) (Dautzenberg et al., 2004). The presence of CRF_2a_ in amphibian species suggests an earliest occurrence of this splice variant during vertebrate evolution, while the CRF_2b_ is less conserved and appears to be evolutionarily younger and is found only in mammals (Dautzenberg et al., 2001). Recently, Chen et al. reported a novel CRF_2a_ splice variant in the mouse brain that includes the first extracellular domain of the CRF_2a_ receptor and acts as a soluble binding protein (sCRF_2a_), thereby modulating the accessibility and signaling (Chen et al., 2005). In the rat esophagus, CRF_2b_ wild-type transcript is predominantly expressed and in addition, several new CRF_2_ splice variants including six CRF_2a_ isoforms were identified (Wu et al., 2007; Yuan et al., 2012b).

Similar to CRF, the CRF_1_ receptor displays a wide distribution throughout the brain. In rats, the CRF_1_ is densely and widely expressed in the forebrain, in the septal region and amygdala (Justice et al., 2008), whereas basal expression is low in the hypothalamus but up-regulated under conditions of stress and also by CRF as a feed-forward mechanism (Bonaz and Rivest, 1998; Imaki et al., 2001; Konishi et al., 2003). Contrasting with the wide distribution of the CRF_1_, expression of the CRF_2_ is mainly found in the lateral septum, amygdala and hypothalamic nuclei including the SON and VMH, dorsal raphe, area postrema and nucleus tractus solitarius (Bittencourt and Sawchenko, 2000; Chen et al., 2012). Both, CRF_1_ (laminae III-VIII) and CRF_2_ mRNA (laminae III-X) were also detected in the mouse spinal cord (Korosi et al., 2007). In the periphery, the CRF_1_ was detected in the anterior and intermediate lobe of the pituitary gland (Turnbull and Rivier, 1997) and in the gastrointestinal tract more prominently in colonic endocrine, neuronal and immune cells (Chatzaki et al., 2004; Yuan et al., 2007, 2012a), while CRF_2_ was found in the stomach including the luminal surface of the crypts and in blood vessels of the submucosal layer (Chatzaki et al., 2004; Yuan et al., 2012b).

Following the identification of CRF receptors, the characterization of binding affinities to endogenous ligands showed that the CRF_1_ and CRF_2_ receptors display distinct affinities to CRF and urocortins (Lewis et al., 2001; Hauger et al., 2003; Hillhouse and Grammatopoulos, 2006). CRF displays a 10- to 40-fold higher affinity for the CRF_1_ than the CRF_2_ receptor, whereas Ucn 1 binds with equal affinity to both CRF receptors thereby displaying a 100-fold higher affinity to the CRF_2_ receptor compared to CRF (Grace et al., 2007). It is important to note that so far no endogenous selective ligand for the CRF_1_ has been identified. In contrast to CRF and Ucn 1, Ucn 2, and Ucn 3 show high selectivity for the CRF_2_ (Grace et al., 2007). In contrast to the CRF_1_ receptor isoforms, binding characteristics of the CRF receptor splice variants, CRF_2a_, CRF_2b_, and CRF_2c_ are almost identical with high affinity for Ucn 1, Ucn 2, and Ucn 3, and lower affinity for r/hCRF (Kostich et al., 1998; Ardati et al., 1999; Suman-Chauhan et al., 1999; Lewis et al., 2001). However, the isoforms of CRF_2a_ show distinct pharmacological profiles; the mouse sCRF_2a_ receptor has very low affinity for Ucn 2 and Ucn 3, while binding to Ucn 1 (Ki 6.6 nM), and, to a lesser extent, to CRF (23 nM), and inhibits the cAMP and ERK1/2-p42,44 responses to Ucn 1 and CRF (Chen et al., 2005). In contrast, rat CRF_2a−tr_ binds with low affinity to CRF (Kd 12.7 nM) and does not bind to Ucn 1 (Miyata et al., 1999).

## Food intake inhibitory actions of brain CRF and Ucns

It is well documented that members of the CRF family and more prominently Ucns injected into the brain ventricle suppress food intake in various species (Zorrilla et al., 2003; Wang et al., 2011b) and increase energy expenditure (Richard et al., 2002). In *ad libitum* fed rats, intracerebroventricular (icv) injection of CRF (Spina et al., 1996), Ucn 1 (Spina et al., 1996; Yakabi et al., 2011), Ucn 2 (Ohata and Shibasaki, 2004), and Ucn 3 (Ohata and Shibasaki, 2004) decreases dark phase food intake. At the lowest icv doses, Ucn 1 and Ucn 2 decrease food intake without inducing conditioned taste aversion or visceral illness (Benoit et al., 2000; Inoue et al., 2003; Zorrilla et al., 2004). It is to note that icv Ucn 1 is more potent in suppressing food intake compared to CRF (Spina et al., 1996), and Ucn 2 is 10-fold more potent than Ucn 3 in reducing food intake (Pelleymounter et al., 2004). Kinetic studies also showed differences in the time course of food intake suppression induced by icv injection of the CRF-related peptides. CRF, a preferential CRF_1_ agonist, induces a rapid onset and short-term reduction of the re-feeding response to an overnight food deprivation in mice and rats (Ohata and Shibasaki, 2004; Pelleymounter et al., 2004), while the dark phase food intake-reducing effect of icv Ucn 2 or Ucn 3 is delayed (onset >3 h) and long lasting in rats (Inoue et al., 2003; Zorrilla et al., 2004). Conversely, in CRF_1_ deficient mice, icv Ucn 1 still induces a delayed onset suppression of food intake while the early phase is no longer observed (Bradbury et al., 2000). Similar to the acute effects, under chronic conditions observed in mice overexpressing CRF there is also a curtailing of the re-feeding response to a fast (Stengel et al., 2009).

Convergent studies established the primary role of CRF_2_ in mediating the anorexigenic effect of acute or repeated icv injections of CRF or Ucns using complementary pharmacologic (selective CRF_1_ or CRF_2_ antagonists) (Smagin et al., 1998; Contarino et al., 2000; Pelleymounter et al., 2000; Cullen et al., 2001; Sekino et al., 2004) and gene deletion (CRF_1_ or CRF_2_ deficient mice) (Bradbury et al., 2000) approaches in addition to the selective CRF_2_ agonists, Ucn 2, and Ucn 3 (Cullen et al., 2001; Richard et al., 2002; Zorrilla et al., 2003; Pelleymounter et al., 2004). To date, the role of CRF_1_ in mediating the effects of icv CRF and Ucn 1 appears to be less specific and is confounded by competing behaviors (increased locomotion, grooming, anxiety- or fear-like) induced by activation of the brain CRF_1_ signaling pathway. This contrasts with icv Ucn 2 that does not elicit behavioral arousal or anxiogenic effects (Pelleymounter et al., 2000; Inoue et al., 2003; Zorrilla et al., 2004; Jochman et al., 2005). Several brain sites expressing high concentrations of CRF_2_ (Bittencourt and Sawchenko, 2000) have been identified to be responsive to Ucns resulting in a CRF_2_-mediated anorexigenic response, namely the lateral septum (Bakshi et al., 2007), PVN (Currie et al., 2001), VMH (Ohata et al., 2000; Fekete et al., 2007; Chen et al., 2012), medial amygdala (Fekete et al., 2007) and dorsal raphe (Weitemier and Ryabinin, 2006). In addition, hindbrain structures are involved based on the observation that Ucn 1 injected into the fourth brain ventricle is still able to reduce food intake in chronically decerebrated rats (Daniels et al., 2004). Among those, the nucleus tractus solitarius has been identified as a brainstem site responsive to Ucn 1 (Grill et al., 2000).

Analysis of changes in feeding patterns associated with the decreased food intake indicates that Ucn 2 injected icv reduced the size of the meal (increased satiation) and the rate of ingestion, whereas meal frequency was not altered in rats (Inoue et al., 2003). In addition, Ucn 3 injected icv, and even more potently, when microinjected into the PVN and VMH, increased inter-meal interval (induction of satiety), whereas meal size was reduced (induction of satiation) at the highest dose only (Fekete et al., 2007). In the medial amygdala, Ucn 3 was shown to promote nibbling (smaller but more frequent meal) (Fekete et al., 2007), indicating the distinct modulation of feeding patterns by Ucn 2 and Ucn 3 and the influence of brain sites of action.

The physiological relevance of the brain CRF_2_ signaling pathways in the regulation of feeding pattern and body weight is supported by reports that CRF_2_ knockout mice showed increased nocturnal food intake of normal chow (Tabarin et al., 2007) and consumed more high fat food compared to their wild type littermates (Bale et al., 2003). In addition, mice with a site specific knockdown of CRF_2_ in the VMH using small hairpin RNA exhibit increased food intake under basal and stimulated conditions by an overnight fast (Chao et al., 2012). This supports a role of CRF_2_ in the VMH to curtail the cessation of eating. There is also evidence that continuous icv infusion of the CRF_2_ antagonist antisauvagine-30 over 13 days increased food intake by 5% in normal rats (Cullen et al., 2001), while chronic injection of a CRF_1_ antagonist had no effect (Ohata et al., 2002). However mice lacking Ucn 1 (Vetter et al., 2002) or Ucn 2 (Chen et al., 2006) have a normal spontaneous food intake which may merely emphasize the compensatory mechanisms by other endogenous peptide members of the CRF family as genetic deficient Ucn 3 showed elevated basal feeding and increased nocturnal food intake after overnight fasting compared with the wild-type littermates (Chao et al., 2012).

Several potential mechanisms could participate in icv Ucns-induced anorexia. Central injection of CRF and Ucns potently suppressed gastric emptying (Stengel and Taché, 2010) and induces hyperglycemia (Brown et al., 1982; Chen et al., 2010). Both effects are known to reduce feeding. Delayed gastric emptying by slowing meal transit leads to accrual of food in the stomach and consecutively to gastric satiety signaling to the brain (Phillips and Powley, 1996) while elevated glucose exerts a direct action on glucose sensing of hypothalamic neurons regulating food intake (Levin, 2006; Cha et al., 2008). There is also evidence Ucn 1 icv suppressed circulating acyl ghrelin (Yakabi et al., 2011), the only known peripherally produced and centrally acting orexigenic hormone (Hosoda et al., 2002; Stengel and Taché, 2012). Moreover, Ucn 1 microinjected into the PVN increased plasma levels of leptin (Kotz et al., 2002), a potent appetite suppressant (Keen-Rhinehart et al., 2013). Future studies are needed to evaluate the relative influence of these hormonal and functional alterations.

Besides the reduction in food intake, accumulated evidence indicates that activation of brain CRF and Ucns signaling pathways increases energy expenditure (Richard et al., 2002; Kuperman and Chen, 2008). Sympathetically-regulated heat production in brown adipose tissue (BAT) and lipid metabolism contributes to the non-shivering thermogenic component of energy expenditure and body weight regulation in rodents (Landsberg et al., 1984). The icv injection of Ucns increases oxygen consumption in rats as assessed by indirect calorimetry and elevates body temperature resulting in increased energy expenditure (De Fanti and Martinez, 2002; Telegdy and Adamik, 2008). Microinjection studies showed that CRF sites of action to increase sympathetic nerve activity to interscapular BAT leading to BAT-mediated thermogenesis and energy expenditure are located in the medial preoptic area and dorsomedial hypothalamus unlike the PVN or VMH (Egawa et al., 1990; Cerri and Morrison, 2006; Chao et al., 2012). However, during food restriction, Ucn 1 microinjected into the PVN increases thermogenic capacity by elevating uncoupling protein-1 mRNA levels in BAT (Kotz et al., 2002). The peptide at this site also changes energy substrate utilization as shown by reduction of the respiratory quotient (QR) under basal or NPY- or ghrelin-stimulated conditions (Currie et al., 2001). The CRF_2_ seems to play a major role in the effects on energy expenditure as mice lacking the CRF_1_ showed a greater reduction in body weight following a 7-day icv infusion of Ucn 1 compared to their wild type littermates (Bradbury et al., 2000). Additionally, the hyperthermic response to icv Ucn 2 and Ucn 3 was blocked by selective CRF_2_ receptor antagonists while CRF_1_ blockade had no effect (Telegdy et al., 2006). Moreover, selective depletion of CRF_2_ in the VMH reduced lipolysis and increased white fat (Chao et al., 2012).

## Food intake inhibitory actions of peripheral CRF and Ucns

In addition to the central actions of CRF and Ucns, peripheral (intraperitoneal, ip) injection of Ucns reduced food intake in several species (Asakawa et al., 1999; Weisinger et al., 2000; Wang et al., 2001; Tanaka et al., 2009) and repeated administration also lowered body weight gain in mice (Asakawa et al., 1999). The anorexigenic effect of Ucn 1 on re-feeding food intake was stronger than that of cholecystokinin (CCK), leptin, and also CRF in mice (Asakawa et al., 1999; Wang et al., 2001; Tanaka et al., 2009). Interestingly, a synergistic interaction between ip injected Ucn 1 and CCK-8 to reduce the feeding response to a fast and liquid gastric emptying has been reported in mice (Gourcerol et al., 2007). The demonstration that Ucn 1 injected peripherally displays a similar potency as after icv injection supports a peripherally initiated mode of action (Cullen et al., 2001; Sinnayah et al., 2003; Pelleymounter et al., 2004). However, it is to note that systemic doses at which the preferential CRF_1_ agonist CRF, and potent CRF_1_/CRF_2_ agonist, Ucn 1 decrease food intake are associated with conditioned taste aversion and diarrhea (Fekete et al., 2011), which are not observed at the lower anorexigenic doses of Ucn 1 given icv (Benoit et al., 2000; Inoue et al., 2003; Zorrilla et al., 2004).

The reduction of food intake by ip Ucn 1 is CRF_2_ mediated as the selective CRF_2_ antagonists, antisauvagine-30 and astressin_2_-B blunted the reduction of food intake, whereas selective CRF_1_ antagonists did not (Weisinger et al., 2000; Wang et al., 2001, 2011b). In line with this finding, fasted mice lacking the CRF_2_ did not show a reduction of re-feeding food intake following ip injected Ucn 1 (Wang et al., 2011b). The mechanisms through which peripheral Ucn 1 exerts its anorexigenic effect do not involve capsaicin sensory afferents, unlike ip CCK-8 tested under the same conditions in mice (Wang et al., 2001). It was also shown that the slowing of gastric emptying associated with ip Ucn 1 accounts only for 35% of the reduction of food intake induced by Ucn 1 (Wang et al., 2001). It may be speculated that Ucn 1 acts through CRF_2_ densely expressed in appetite-/taste aversion-regulating brain structures outside the blood brain barrier, namely the area postrema (Sakai and Yamamoto, 1997; Bittencourt and Sawchenko, 2000) shown to be activated by systemic injection of Ucn 1 (Wang et al., 2000). This contention will need to be further ascertained.

With regards to Ucn 2 and Ucn 3, both peptides induce a rapid in onset and CRF_2_ mediated reduction of re-feeding food intake after a fast and also dampen *ad libitum* dark phase food intake with a potency of Ucn 2 > Ucn 3 in rodents (Wang et al., 2001, 2011b; Gourcerol et al., 2007; Tanaka et al., 2009; Fekete et al., 2011). In contrast to ip Ucn 1, Ucn 2 given ip at an anorexigenic dose did not induce signs of taste aversion or malaise (Fekete et al., 2011). Of interest, there is a synergistic interaction between ip CCK-8 and Ucn 2 to reduce the re-feeding response to a fast in mice which was also observed at the level of vagal activity recorded from the gastric afferents in an *in vitro* preparation (Gourcerol et al., 2007). This functional and electrophysiological evidence combined with the expression of CRF_2_ in rat nodose ganglia (Mercer et al., 1992; Lawrence et al., 2002) point toward a role of vagal signaling in the mediation of ip Ucn 2 anorexic action that will need to be ascertained using vagal deafferentation.

Additional insight into the characterization of the food intake-reducing effects of peripheral administration of Ucns came from studies detailing changes in meal patterns. Using the micropellet technique, ip injection of Ucn 1 reduces the meal frequency (as a characteristic of satiety), whereas the size of the meal (as a characteristic of satiation) was less robustly altered during the re-feeding period following a 24-h fast in rats (Fekete et al., 2011). Under the same conditions, ip injection of Ucn 2 only reduced meal frequency while not altering meal size (Fekete et al., 2011). These data were recently extended to mice following ip injection of Ucn 2 using an automated episodic food intake monitoring system for solid food: Ucn 2 reduced meal size and duration (induction of satiation) but also increased meal frequency (reduction of satiety) in overnight fasted mice (Wang et al., 2011b). Interestingly, when injected ip in freely fed animals at the beginning of the dark phase, Ucn 2 only affected satiation (reduction of meal size), whereas satiety (indicated by the number of meals) was not altered (Wang et al., 2011b) giving rise to a differential modulation of light and dark phase feeding by Ucn 2.

## Involvement of brain CRF signaling pathways in the anorexigenic response to stress

Accumulated evidence supports a role of brain activation of CRF signaling pathways in initiating the hypophagia and weight loss induced by various types of stressors. This was established using pharmacological approaches and to a lesser extent, genetic deletion of CRF ligand or receptors in rodents. Earlier studies showed that icv or 3rd ventricular injection of the non-selective CRF_1_/CRF_2_, antagonist, α-helical CRF_9−41_ before exposure to stressors prevented the decreased food intake observed after acute exposure to restraint (Krahn et al., 1986; Shibasaki et al., 1988; Smagin et al., 1999), 40 min forced exercise (Rivest and Richard, 1990) or emotional stress induced by a communication box paradigm (Hotta et al., 1999) while having no effect on food intake under non-stressful conditions (Hotta et al., 1999). The CRF antagonist injected into the 3rd ventricle also blocked repeated restraint-induced weight loss (Smagin et al., 1999). Subsequent studies to elucidate the importance of each CRF receptor subtype in the stress-related decrease in food intake indicated that either CRF receptor subtype alone or in combination can be involved in the hypophagia depending upon the modality of the stressors. Evidence so far supports that the relative ability of selective CRF receptor subtype antagonists to block stress-related anorexia is critically dependent upon specific brain sites activated and related CRF ligands and receptor subtypes recruited by different stressors. For instance, emotional stress triggered by the communication box paradigm, foot shock and 1-h restraint was reported to involve both CRF_1_ and CRF_2_ receptors in rats as shown by partial or complete reversal induced by either icv injection of CRF_2_ antagonist, antisauvagine-30 or peripheral administration of the CRF_1_ antagonist, CRA 1000 (Hotta et al., 1999; Sekino et al., 2004). In mice, novelty/group separation stress-induced reduction of food intake was curtailed by icv pretreatment with the CRF_1_ antagonist, NBI- 127914 while the potent CRF_2_ antagonist, astressin_2_-B had no effect (Saegusa et al., 2011). Brain site specific targeting by CRF antagonists indicates that the CRF_1_ antagonist, NBI-27914 microinjected into the basolateral amygdala, unlike central amygdala, prevented emotional stress (rat exposed to predator)-induced decreased food intake and increased grooming while astressin_2_-B had no effect (Jochman et al., 2005). By contrast, the CRF_2_ antagonist, antisauvagine-30 microinjected into the lateral septum or posterior division of the bed nucleus of the stria terminalis prevented acute restraint-induced anorexia while the selective CRF_1_ antagonist, antalarmin had no effect in rats (Ohata and Shibasaki, 2011). There is also evidence that CRF_2_ knockout mice showed an abbreviated inhibition of food intake induced by restraint stress while not being involved in the early orexigenic response (Tabarin et al., 2007).

Of (patho)physiological relevance is the demonstration that mutation in the CRF_2_ gene (Val411Met) was associated with an early onset of severe obesity (Challis et al., 2004). Moreover, several studies observed an association with a portion of chromosome 7 also coding the CRF_2_ gene (7p15–7p21) and body mass index (Wu et al., 2002), type 2 diabetes mellitus (Wiltshire et al., 2001) and also fat-free body mass (Chagnon et al., 2000). Future studies are needed to corroborate these findings and investigate the consequences of these associations.

## Summary

In summary, activation of CRF_2_ by Ucns reduced feeding after central as well as peripheral injection without provoking behavioral arousal or anxiogenic effects as observed for the anorexigenic action induced by the activation CRF-CRF_1_ signaling pathways. Brain sites of action of Ucns involve the lateral septum, PVN, VMH, medial amygdala, dorsal raphe and nucleus tractus solitarius. Both, CRF_1_ and CRF_2_ receptor activation contributes to the reduction of food intake associated with exposure to various stressors. Their respective involvement is stressors and brain sites specific mostly in relation with endogenous CRF ligands and receptors recruited under the condition of stress. Still little is known whether peripheral Ucn-CRF_2_ signaling plays a role in the food intake response to visceral stressors. More research is needed using site specific knockout or overexpression of CRF receptors in order to address this issue and investigate the impact on food intake and body weight.

### Conflict of interest statement

The authors declare that the research was conducted in the absence of any commercial or financial relationships that could be construed as a potential conflict of interest.
